# Protective immunity against *Shigella sonnei* in vaccinated and challenged adult Thai volunteers

**DOI:** 10.1128/msphere.00269-26

**Published:** 2026-07-30

**Authors:** Esther Ndungo, Dilara Islam, Siriphan Gonwong, Nattaya Ruamsap, Patricia L. Milletich, Alain Agnememel, Samandra T. Demons, Marcela F. Pasetti

**Affiliations:** 1 Center for Vaccine Development and Global Health, University of Maryland School of Medicine 12264, Baltimore, Maryland, USA; 2 Armed Forces Research Institute of Medical Sciences (AFRIMS) 544411, Bangkok, Thailand; University of Wyoming College of Agriculture Life Sciences and Natural Resources, Laramie, Wyoming, USA

**Keywords:** *Shigella*, antibodies, vaccines, WRSS1, correlates of protection

## Abstract

A safe, effective, and affordable vaccine that can prevent *Shigella*-induced diarrhea could have a significant impact on reducing morbidity and mortality in populations at risk. WRSS1, a live attenuated *Shigella sonnei* vaccine candidate, was well tolerated and immunogenic in adult Thai volunteers, achieving 40% efficacy against wild-type (WT) *S. sonnei*. We performed an in-depth analysis of mucosal and systemic antibodies in these individuals following WRSS1 vaccination and *S. sonnei* challenge, including a broader analysis of antibody specificity, functional features, and associations with clinical protection. IgG and IgA against *Shigella* proteins IpaB, IpaC, IpaD, IpaH, VirG, and LPS from multiple strains, as well as complement-mediated bactericidal and opsonophagocytic killing activity, were assessed in serum, fecal extracts, and antibodies in lymphocyte supernatants. *Shigella-*specific serum and ALS IgG and IgA increased after WRSS1 vaccination and *S. sonnei* challenge, particularly in naïve individuals, and most robustly post-challenge. Shedding the vaccine or infecting strain was associated with ALS responses. Fecal IgA was broadly reactive to most antigens, while fecal IgG was specific for *S. sonnei* LPS. Functional antibodies were detected prominently in fecal extracts; the highest responders were naïve individuals post-challenge. Notably, higher protein-specific serum IgA titers before challenge were associated with clinical protection against disease. Our results highlight the nuanced immunity to *Shigella* in endemic regions and the importance of understanding the elements that mediate protection in these settings to inform effective vaccine implementation.

**IMPORTANCE:**

Understanding the immune response in *Shigella*-endemic regions is critical for the design and implementation of vaccines for the target population. We characterized the breadth of systemic and mucosal antibody responses to WRSS1 vaccination and WT *S. sonnei* challenge in Thai adult volunteers. Antigen-specific IgG and IgA in ALS were elevated only in individuals who shed the vaccine or challenge strain, indicating exposure. Serum IgA responses to *Shigella* proteins were associated with clinical protection. Our findings underscore the value of serosurveillance in guiding public health interventions and support the concept of protein-based vaccines to prevent disease in high-burden settings.

## INTRODUCTION

Children living in low- and middle-income countries are disproportionately affected by *Shigella*-induced diarrhea; all ages are at risk, but the most afflicted are children under 5 years of age who account for >60,000 deaths annually (1). Other vulnerable groups include travelers and military personnel deployed to endemic regions (2). *Shigella* outbreaks have also been reported periodically in industrialized countries (1–3). Of the four major species, *Shigella flexneri* and *Shigella sonnei* are the most prevalent worldwide (2, 4, 5). The global impact of *Shigella* disease (shigellosis) and the rapid increase in multidrug resistance compel the development of control and treatment options as public health priorities (2).

Understanding host immune responses that can prevent *Shigella* infection and identifying immunological correlates that predict vaccine efficacy in the target population are essential for advancing vaccine development. Serum IgG against the *Shigella* lipopolysaccharide (LPS) has been associated with reduced risk of illness in outbreaks and field trials (6, 7). Controlled human infection models (CHIMs) in North American individuals have uncovered strong correlations between protection against infection and serum IgG specific for *Shigella* invasion plasmid antigens IpaA, IpaB, and IpaC, as well as the virulence antigen VirG (8–10). Functional antibodies with bactericidal (BA) and opsonophagocytic killing activity (OPKA) in serum have also been evaluated as predictors of protective immunity and vaccine efficacy (8, 11–14).

Mucosal immunity has garnered attention as crucial in preventing *Shigella* infection. IgG and IgA in fecal extracts, as well as the frequency of mucosally primed LPS-specific antibody-secreting cells (ASC) and antibodies in lymphocyte supernatants (ALS), are considered putative indicators of protective immunity (15–19). Although protective biomarkers have been identified in certain cohorts, results remain inconsistent across studies, and firm immunological correlates have yet to be established. Of particular interest are predictors of vaccine efficacy in endemic regions.

Among the various strategies proposed to prevent shigellosis, WRSS1 stands out as a live, attenuated oral *S. sonnei* vaccine candidate that harbors a deletion of the virulence plasmid-encoded VirG (IcsA), which is essential for intercellular spread (20). WRSS1 was immunogenic but reactogenic in U.S. individuals in a phase 1 study (21). However, WRSS1 was well tolerated in individuals living in *Shigella*-endemic areas, including Israeli adults (22) and Bangladeshi adults and children (23). In healthy Thai adults, WRSS1 given as a single oral dose of 1.6 × 10^4^ colony-forming units (CFU) was safe and afforded 40% vaccine efficacy after challenge with 1.67 × 10^3^ CFU of wild-type (WT) *S. sonnei* 53G (15). An initial analysis of immune responses from that study focused on *S. sonnei* LPS-specific antibodies in serum, ALS, and fecal extracts, as well as on *S. sonnei* LPS-specific ASC (15, 24). Neither immunity against other important bacterial antigens nor antibody function was assessed.

In this study, we report an in-depth analysis of humoral immunity in Thai volunteers who were vaccinated with WRSS1 and subsequently challenged with *S. sonnei*. We assessed binding antibodies against a broad antigenic repertoire and functional antibodies in systemic and mucosal samples. IgG and IgA against *Shigella* IpaB, IpaC, IpaD, IpaH, VirG, *S. flexneri* 2a LPS, *S. flexneri* 3a LPS, *S. flexneri* 6 LPS, and *S. sonnei* LPS were quantified in serum, lymphocyte supernatants, and fecal extracts using a multiplex platform (25). *S. sonnei-*specific functional antibodies were also measured in serum, lymphocyte supernatants, and fecal extracts using quantitative BA and OPKA assays. The identified antibody responses were compared with shedding of vaccine and challenge strains and with clinical outcomes post-infection.

## RESULTS

### Cohort characteristics and clinical specimens

**Fig 1:**
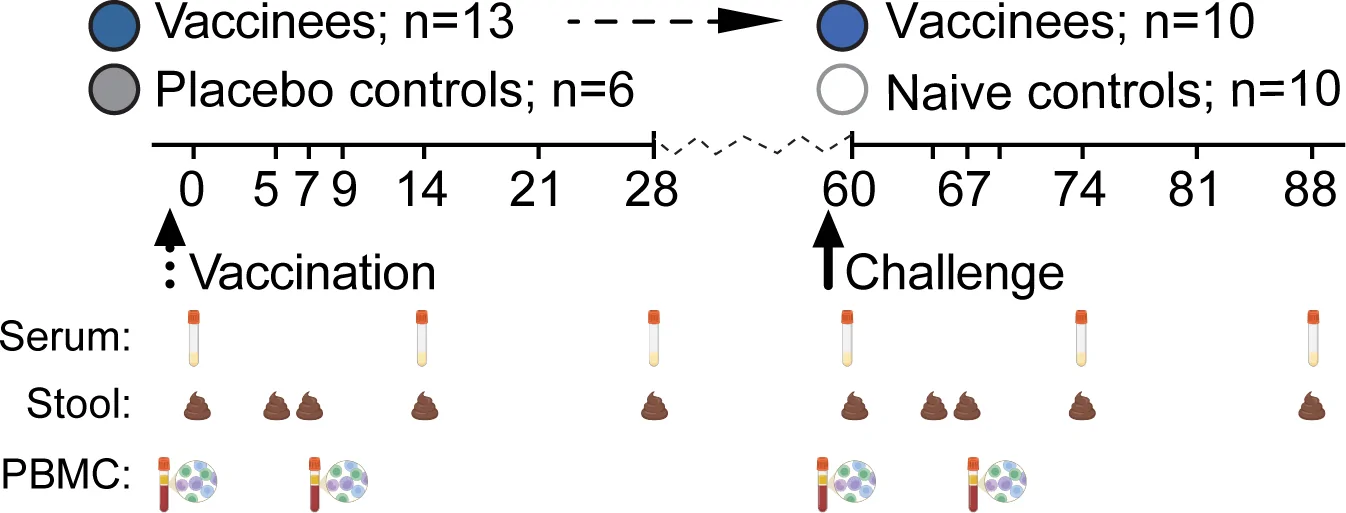
WRSS1 vaccination and challenge study design and specimen collection. Thai adult individuals received a single oral dose of WRSS1 or placebo (placebo controls). On day 60, study participants who had been vaccinated with WRSS1 and a new group of unvaccinated individuals (naïve controls) were challenged with wild-type *S. sonnei* 53G. Sera and stools were collected before vaccination (day 0) and challenge (day 60) and again 2 and 4 weeks after vaccination (days 14 and 28) and challenge (days 74 and 88). Stool was also collected early after dosing, i.e., on days 5 and 7 post-vaccination and challenge. Peripheral blood mononuclear cells (PBMCs) were obtained on days 0 and 9 after vaccination or challenge for measurement of antibody in lymphocyte supernatant (ALS).

Serum, fecal extracts, and lymphocyte supernatants were obtained from Thai adults who participated in a randomized, double-blind, placebo-controlled study evaluating the safety, immunogenicity, and efficacy of WRSS1 (15). In the first phase of the study, participants received WRSS1 as a single oral dose of 1.6 × 10^4^ CFU or placebo (*n* = 13 and 6, respectively). In the second phase, vaccine efficacy was assessed by challenging randomly selected vaccine recipients from the first phase (*n* = 10) and a group of newly recruited naïve volunteers (*n* = 10) with 1.67 × 10^3^ CFU of wild-type *S. sonnei* 53G (15). A schematic representation of the study design and specimen collection is shown in Fig. 1.

IgG and IgA against *Shigella* proteins IpaB, IpaC, IpaD, IpaH, and VirG and against the LPS from *S. sonnei* and other diarrheagenic serotypes for *S. flexneri* 2a, *S. flexneri* 3a, and *S. flexneri* 6 were quantified using a Meso Scale Discovery (MSD) electrochemiluminescence-based multiplex assay (25). The functional capacity of *S. sonnei*-specific antibodies, bactericidal and opsonophagocytic killing, was also examined. Antibody responses were calculated as the geometric mean fold rise (GMFR) in antibody titers after vaccination or challenge relative to baseline (day 0 [pre-vaccination] for the first phase and day 60 [pre-challenge] for the second phase). Response rates were defined as the proportion of individuals in each group who demonstrated a ≥4-fold increase in antibody levels on any day post-vaccination or post-challenge.

### Antibody responses following WRSS1 vaccination

**Fig 2:**
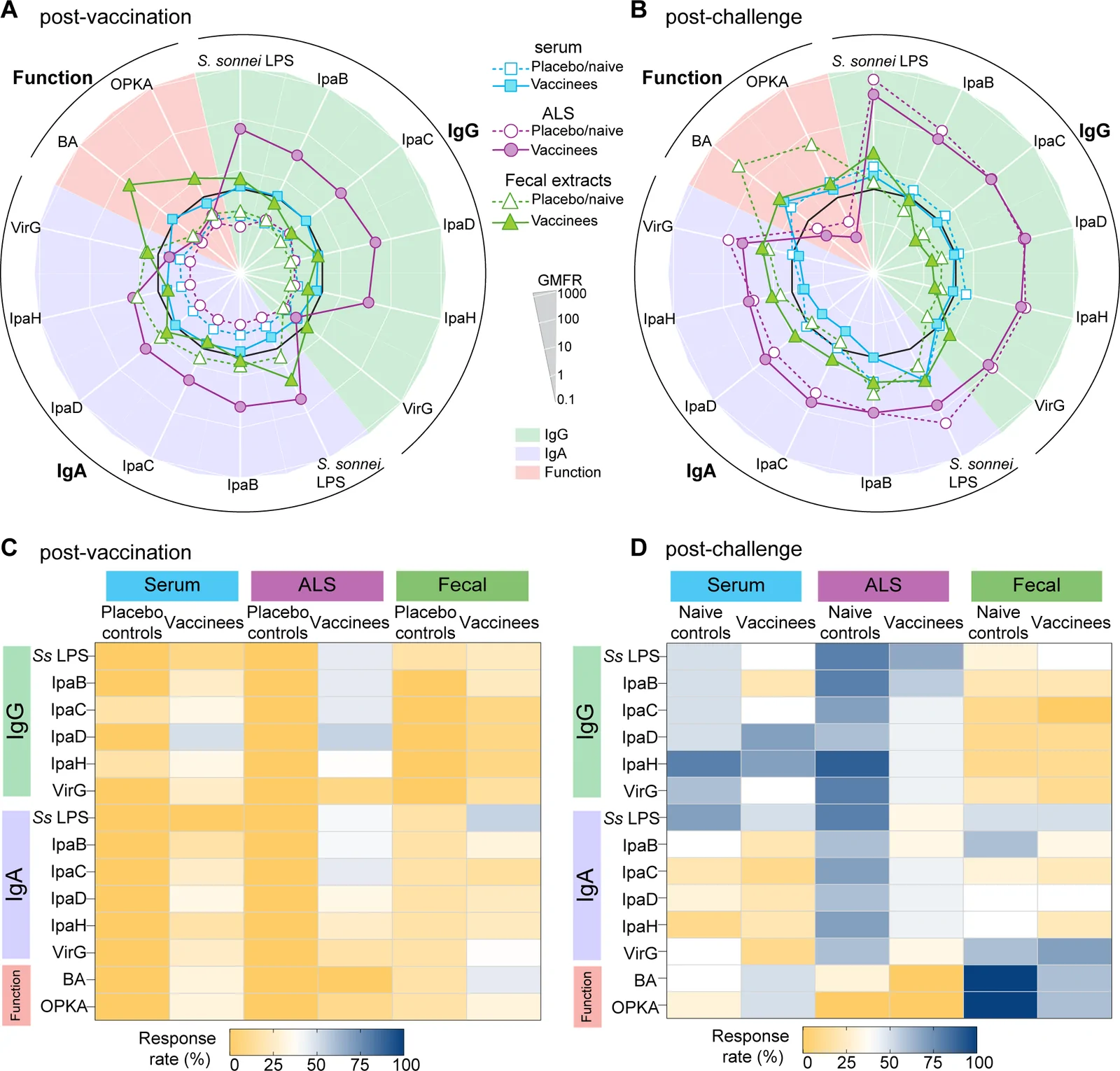
*Shigella* antigen-specific antibody levels following WRSS1 vaccination and *S. sonnei* challenge. Radar plots depict geometric mean fold rise (GMFR) of antigen-specific IgG, IgA, and complement-dependent antibody-mediated function: bactericidal (BA) and opsonophagocytic activity (OPKA) measured in serum (blue squares), ALS (purple circles), and fecal extracts (green triangles) from (**A**) WRSS1 vaccinees or placebo controls post-vaccination and (**B**) WRSS1 vaccinees or naïve controls post *S. sonnei* challenge. Symbols represent peak GMFR (the maximum at any time point after vaccination or challenge). The black solid line represents a fourfold GMFR over baseline (day 0 or 60). Heatmaps depict the percentage of individuals in each group that responded (≥4-fold GMFR over baseline) post-vaccination (**C**) or post-challenge (**D**). *Ss*, *S. sonnei*.

WRSS1 vaccination resulted in increased levels of antigen-specific IgG, IgA, and functional antibodies in serum, lymphocyte supernatants, and fecal extracts (Fig. 2A and Table S1 through S4). ALS had the highest overall response compared to serum and fecal extracts (Fig. 2A), with ALS *S. sonnei* LPS IgG and IgA showing the strongest increase (day 9 GMFRs = 64.1 and 51.6, respectively) (Fig. 2A and Table S2). The ALS IgG responses for IpaB, IpaC, IpaD, and IpaH were higher than the IgA responses (GMFR: IgG = 31.6–49.3, IgA = 13.7–39.2) (Table S2). ALS responses to VirG were marginal (GMFR: IgG = 2.4, IgA = 2.6) (Fig. 2A and Table S2), consistent with the *virG* gene deletion in WRSS1 (20). Functional antibody responses in lymphocyte supernatants were negligible, with GMFR <2 for both BA and OPKA.

In contrast, BA and OPKA responses were strongest in fecal extracts, attaining GMFRs of 57.4 and 11.3, respectively, 2 weeks after vaccination (Fig. 2A and Table S3a), while GMFRs in the placebo recipients ranged between 1 and 1.9 at all time points measured. *S. sonnei* LPS-specific IgA responses peaked earlier (2 weeks post-vaccination) and were higher (GMFR = 19.5) compared with IgG responses, which peaked at 4 weeks (GMFR = 7.0). Fecal IgA vaccine responses against IpaB (peak GMFR = 4.9 at day 28) and IpaD (peak GMFR = 6.7 at day 14), and IgG and IgA against VirG (peak GMFR of 4.6 and 7.0 at days 28 and 14, respectively) were likewise observed (Table S3a).

Serum antibody responses following WRSS1 vaccination were modest, with only a handful of antibody features attaining ≥4-fold GMFR 28 days post-vaccination: *S. sonnei* LPS IgG, IpaB IgG, IpaC IgG, IpaD IgA, and serum BA (Fig. 2A and Table S1). Notably, *S. sonnei* LPS-specific serum IgA responses were low (GMFR = 2.4), despite the increase in IgA levels observed in lymphocyte supernatants and fecal extracts (Fig. 2A). Serum BA responses (peak GMFR = 4.9 at day 14) were also documented (Fig. 2A and Table S1).

Individuals in the placebo group did not show responses (GMFR <2) to any of the antigens in serum or lymphocyte supernatants (Table S1 and S2). No BA and OPA responses were observed in the placebo recipients at any time point in any of the types of specimens tested. One placebo control exhibited elevated fecal IgA responses to all antigens, skewing the overall group response (GMFR ≥4 on day 28), likely due to environmental exposure to *Shigella* in this region (26) (Table S3a).

Despite the increases in antibody levels described above, vaccine response rates were moderate overall and varied by compartment (i.e., systemic or mucosal) or type of sample examined (i.e., serum, ALS, and stool) (Fig. 2C). Most vaccinated individuals (≥50%) had a positive response for IpaD IgG in serum and lymphocyte supernatants, and for *S. sonnei* LPS IgA in fecal extracts (Fig. 2C).

### Antibody responses following WT *S. sonnei* challenge

Antibody responses were markedly stronger and broader after challenge than after vaccination (Fig. 2B and D). Similar to post-vaccination trends, ALS had the largest increases in IgG and IgA after challenge in both naïve participants (60%–90% positive response rate) and those previously immunized (33%–67%) (Fig. 2B and D). ALS responses to *S. sonnei* LPS after challenge were greater in the naïve controls (GMFR: IgG = 594.7, IgA = 177.0) compared to the vaccinated individuals (GMFR: IgG = 303.7, IgA = 71.9) (Fig. 2B and Table S2). Both the naïve and vaccinated participants exhibited robust ALS IgA and IgG responses to the *Shigella* proteins, with comparable magnitudes (naïve GMFR: IgG = 88.0–120.9, IgA = 25.0–79.5; vaccinated GMFR: IgG = 73.5–109.0, IgA = 31.0–61.6) (Fig. 2B). Functional ALS responses were again the lowest following challenge, with GMFR ≤2.5 (Fig. 2B and Table S2).

Post-challenge serum IgG response rates to *Shigella* proteins among naïve individuals ranged from 50% to 70%; the magnitude of responses was similar at 2 and 4 weeks post-challenge, with GMFRs ranging from 3.9 to 7.1 across all antigen specificities (Fig. 2B and Table S1). On the other hand, the target antibody repertoire was narrower among the vaccinated individuals; only IpaD- and IpaH-specific IgG showed majority seroconversion (≥50%), with GMFRs of 4.1 and 3.9, respectively (Fig. 2B and D). The most potent serum IgG response was against *S. sonnei* LPS; 50% of naïve individuals seroconverted (peak GMFR = 11.9) compared with 40% among those previously immunized (peak GMFR = 7.9, both at day 14 post-challenge) (Fig. 2B and D and Table S1). Serum IgA response rates were low overall except for *S. sonnei* LPS, which reached 70% seroconversion in the naïve group and 50% in the immunized group, attaining GMFRs of 22.0 and 21.2, respectively (Fig. 2D). Notably, the naïve controls developed robust serum IgA responses to the *Shigella* proteins, reaching a peak GMFR of 13.2 for IpaB and ≥4-fold GMFRs for IpaC, IpaD, and VirG, while the vaccine recipients responded only to IpaB IgA (GMFR = 4.4). All these serum IgA responses were observed 2 weeks after challenge.

In fecal extracts, more than half of the participants, from either the naïve or immunized group, responded with IgA against *S. sonnei* LPS (Fig. 2D), though the immunized group developed a more robust response (GMFR: IgG = 22.3, IgA = 20.7) than naïve individuals (GMFR: IgG = 6.0, IgA = 9.9) (Fig. 2B and Table S3b). Additionally, the immunized group had a robust protein-specific IgA response, with GMFRs >4 across antigens, whereas naïve individuals developed IgA responses only to IpaB, IpaH, and VirG (GMFR: 4.2–22.2) (Fig. 2B and Table S3b). IpaB-specific fecal IgA was detected at all post-challenge time points in both naïve and immunized groups, with peak response rates of 60% at 7 days post-challenge and 33% at 5 days post-challenge, respectively; GMFRs ranged from 8.4 to 22.2 with a trend toward higher GMFRs in naïve individuals (Table S3b). VirG-specific stool IgA was detected at most post-challenge time points, with peak response rates 7 days after challenge (naïve = 70%, vaccinated = 60%); however, the vaccinated group generally had higher GMFRs than the naïve group (11.4–16.8 vs 3.6–5.5). Protein-specific fecal IgG responses were modest; only immunized individuals mounted responses to IpaB (peak GMFR = 4.0 at 2 weeks post-challenge) and VirG (peak GMFR = 8.1 at 5 days post-challenge) (Fig. 2B and Table S3b).

Functional antibody responses post-challenge were again the highest in fecal extracts, reaching a peak GMFR of 228.6 2 weeks after challenge, vastly exceeding those in lymphocyte supernatants (GMFR = 2.5) and in serum (GMFR = 16.9) (Fig. 2B and Table S3b). All naïve individuals developed an antibody-mediated functional response following challenge, whereas slightly more than half (60%) of vaccinees did (Fig. 2B). Notably, similar to serum and ALS, naïve individuals had higher BA and OPKA responses in fecal extracts compared to the previously vaccinated.

### Antibody responses to non-vaccine LPS antigens

**Fig 3:**
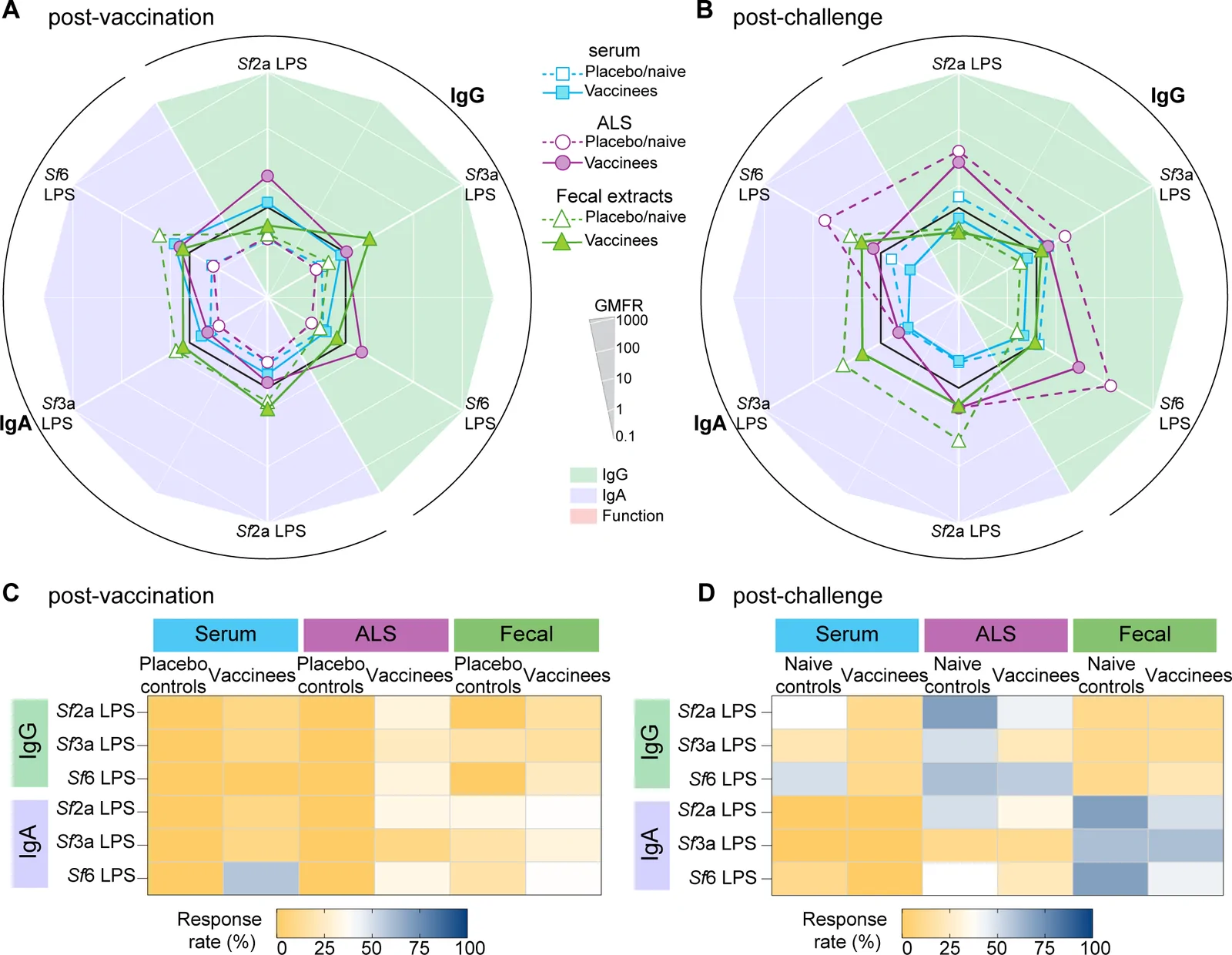
Antibody responses to heterologous *Shigella* LPS following WRSS1 vaccination and *S. sonnei* challenge. Radar plots depict geometric mean fold rise (GMFR) of IgG and IgA against heterologous *Shigella* LPS, i.e., not contained in the vaccine or challenge strains, measured in serum (blue squares), lymphocyte supernatants (purple circles), and fecal extracts (green triangles) from (**A**) WRSS1 vaccinees or placebo controls post-vaccination and (**B**) WRSS1 vaccinees or naïve controls post-*S. sonnei* challenge. Symbols represent peak GMFR (the maximum at any time point after vaccination or challenge). The black solid line represents fourfold GMFR over baseline (day 0 or 60). Heatmaps depict the percentage of individuals in each group who responded (≥4-fold GMFR over baseline) post-vaccination (**C**) or post-challenge (**D**). *Sf*2a, *S. flexneri* 2a; *Sf*3a, *S. flexneri* 3a; *Sf*6, *S. flexneri* 6.

Interestingly, antibody responses (GMFRs ≥ 4) against additional *Shigella* LPS strains (*S. flexneri* 2a, *S. flexneri* 3a, and *S. flexneri* 6) were also observed both after vaccination and challenge (Fig. 3). Post-vaccination, ALS IgG was positive for all strains: GMFRs = 14.4, 4.2, 8.6 for *S. flexneri* 2a LPS, *S. flexneri 3a* LPS, and *S. flexneri* 6 LPS, respectively (Fig. 3A and Table S4b). Similarly, positive fecal IgA responses were observed post-vaccination for all three strains, with peak GMFRs of 9.4, 5.4, and 5.5 for *S. flexneri* 2a LPS, *S. flexneri 3a* LPS, and *S. flexneri* 6 LPS, respectively (Table S4c). Post-vaccination responses were also seen in serum IgG (GMFR = 4.9) against *S. flexneri* 2a LPS, serum IgA (GMFR = 8.2), and ALS IgA (GMFR = 6.3) against *S. flexneri* 6 LPS and fecal IgG (GMFR = 12.6) against *S. flexneri* 3a LPS (Table S4a through c). However, seroconversion rates were <50% except for serum IgA against *S. flexneri* 6 LPS (Fig. 3C). One placebo recipient had fecal IgA responses to all three heterologous LPS antigens 28 days post-vaccination (Table S4c).

Stronger reactivity to heterologous LPS molecules was observed post-challenge, particularly in the naïve individuals (Fig. 3B). In the naïve group, serum IgG responses against *S. flexneri* 2a, *S. flexneri* 3a, and *S. flexneri* 6 LPS were detected with peak GMFR ranging from 4.5 to 7.0; additionally, 50% of the naïve group seroconverted for *S. flexneri* 6 LPS IgG (Fig. 3D and Table S4a). Serum IgG and IgA responses in the vaccinated group were modest (GMFRs: 1.0–2.6).

ALS IgG responses to all three LPS strains were seen in 50%–70% of naïve individuals after challenge (GMFRs: 15.5–134.6) (Fig. 3D and Table S4b). Among those immunized, 56% of individuals exhibited post-challenge ALS IgG responses to *S. flexneri* 6 LPS (GMFR = 29.6) (Fig. 3B and D and Table S4b). ALS IgA responses post-challenge were strongest to *S. flexneri* 2a (naïve: GMFR = 9.1, vaccinees: GMFR = 9.1) and *S. flexneri* 6 LPS (naïve: GMFR = 56.6, vaccinees: GMFR = 5.7) (Fig. 3B and Table S4b). A majority of naïve individuals had robust fecal IgA responses to all three LPS antigens, with peak GMFRs ranging from 16.9 to 33.9, exceeding their responses to *S. sonnei* LPS (peak GMFR = 9) (Fig. 3D and Table S3b and S4d). In contrast, fecal IgA responses in the vaccinated group (peak GMFRs from 8.2 to 9.9) were lower than those to *S. sonnei* LPS (peak GMFR = 20.7) (Fig. 2B and 3B and Tables S3b and S4b). Fecal IgG responses were observed only to *S. flexneri* 3a LPS in vaccinated individuals (Fig. 3B and Table S4d).

### Association between fecal shedding and antibody responses

**Fig 4:**
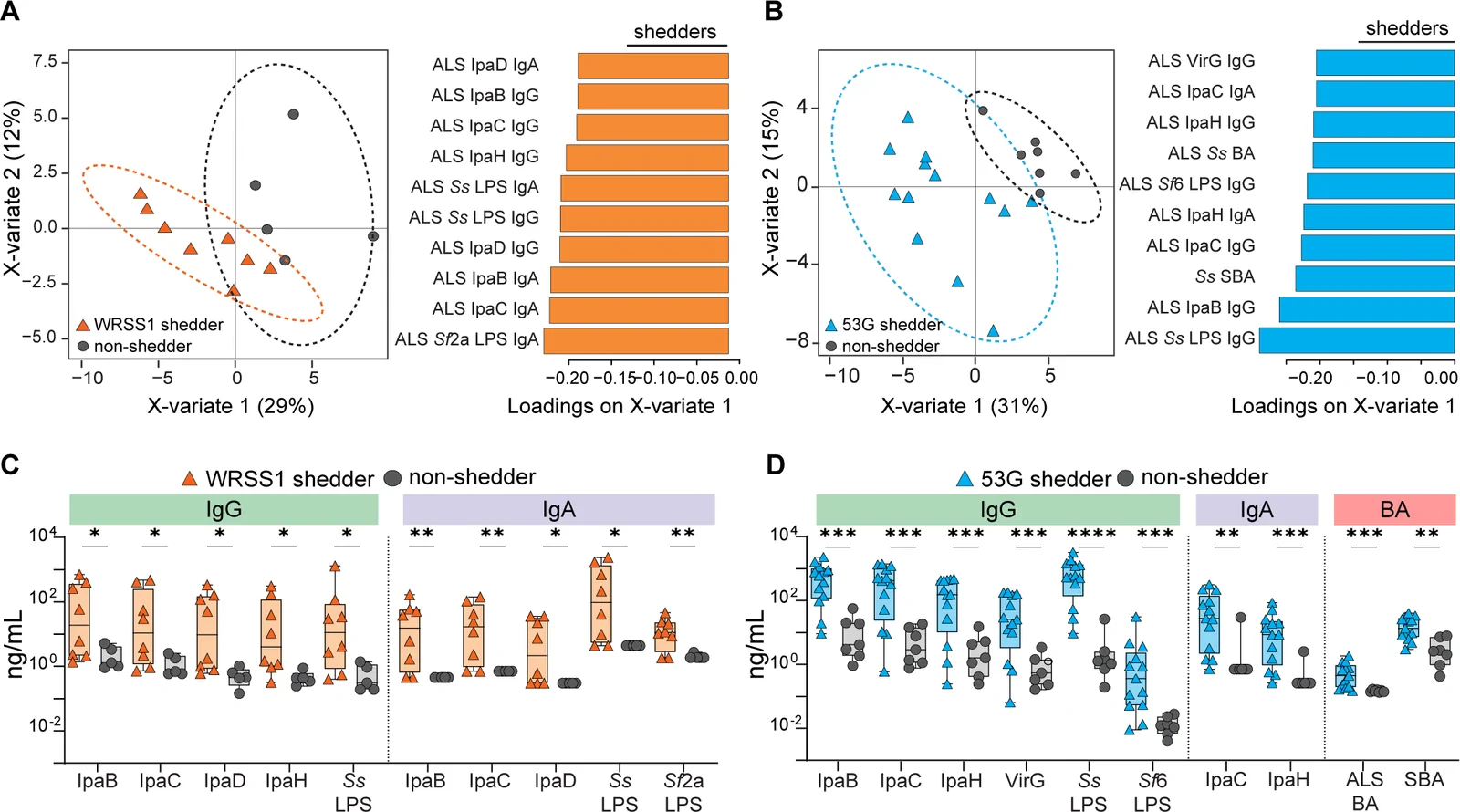
Vaccine- and challenge-strain shedding is associated with stronger ALS responses. PLSDA analysis depicting separation of peak antibody responses (maximum at any time point after vaccination or challenge) between individuals who shed the WRSS1 vaccine strain (**A**) or the WT *S. sonnei* 53G challenge strain (**B**) compared to non-shedders. Bar graphs show immune parameters that contributed significantly to X-variate 1, ranked by loading score, for shedding the vaccine (**C**) or challenge (**D**) strain. Asterisks indicate statistically significant (*P* value < 0.05) differences in log-transformed antibody concentrations of individual shedders and non-shedders. *, *P* < 0.05; **, *P* < 0.01; ***, *P* < 0.001; ****, *P* < 0.0001, as determined by unpaired *t*-test with Welch’s correction.

Previous studies identified positive associations between bacterial shedding and immune responses following oral ingestion of live *Shigella* vaccines, including WRSS1 (21, 27) and challenge (15). To identify antibody features associated with shedding of the WRSS1 vaccine or WT *S. sonnei* challenge strain, we performed a partial least squares discriminant analysis (PLSDA) on all 42 immune parameters measured following vaccination (Fig. 4A) and challenge (Fig. 4B). This analysis was conducted using the peak concentration for each antibody feature, defined as the highest level observed at any post-vaccination or post-challenge time point, and shedders were defined as individuals who were positive for organisms in feces by qPCR (15).

There was a clear separation between antibody features associated with shedding of either the vaccine (Fig. 4A) or the challenge strain (Fig. 4B). ALS IgG and IgA responses were among the top 10 features that discriminated shedders and non-shedders in both post-vaccination and post-challenge (Fig. 4A and B). Post-challenge, ALS and serum BA were also among the top discriminating features (Fig. 4B). Shedders developed significantly higher ALS IgG and IgA titers than non-shedders following vaccination or challenge (Fig. 4C and D).

### Identification of correlates of protective immunity

**Fig 5:**
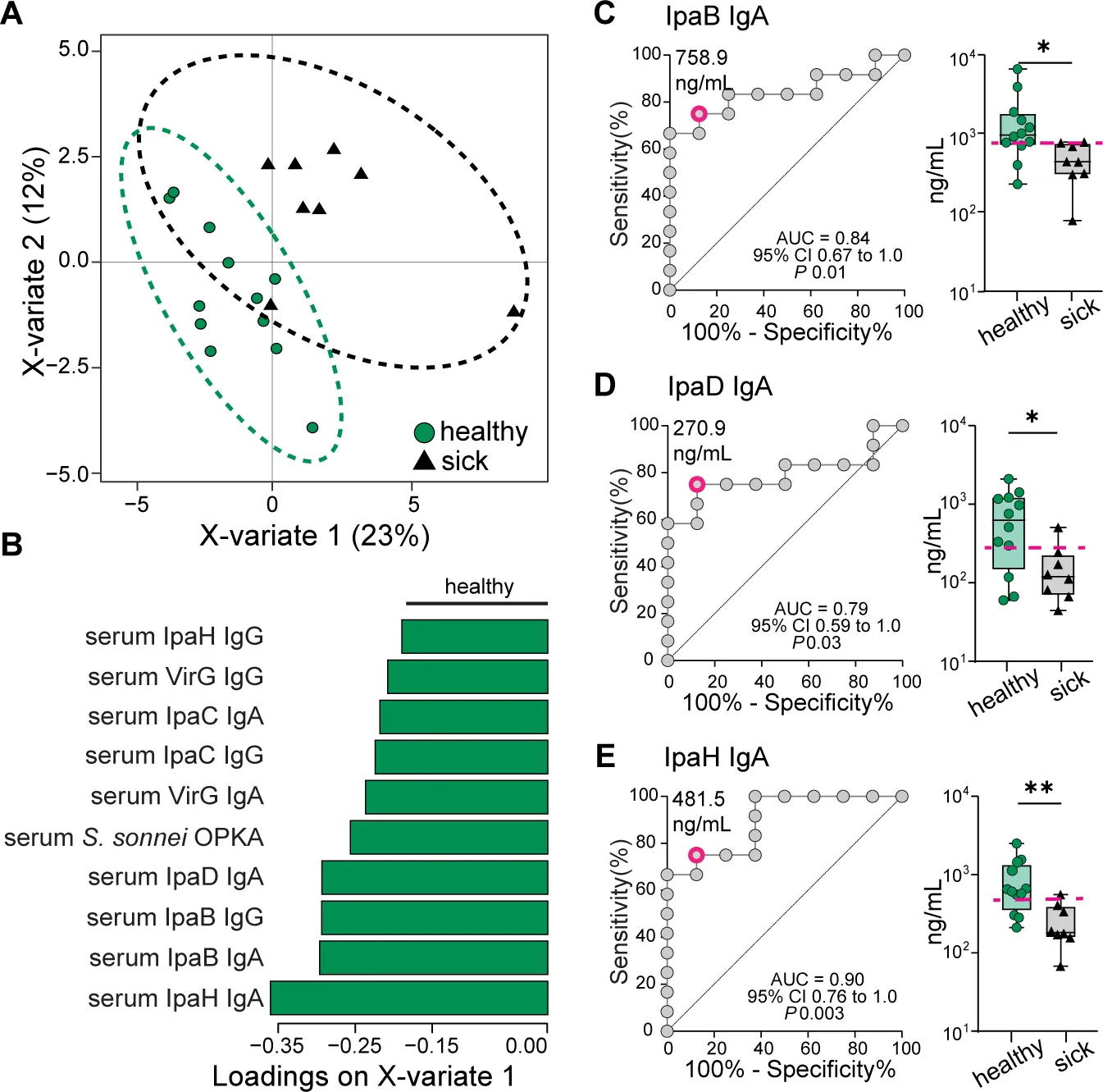
Protein-specific serum IgG and IgA are associated with protective immunity in Thai volunteers. (**A**) PLSDA analysis depicting separation of pre-challenge (day 60) serum antibodies between individuals who remained healthy vs those who became sick after *S. sonnei* challenge. (**B**) Bar graphs show immune parameters in healthy individuals, ranked by loading score, that contributed significantly to X-variate 1. Individual antibody titers in healthy and sick volunteers and receiver operating characteristic curves showing sensitivity vs 100% - specificity% calculated using pre-challenge IpaB serum IgA (**C**), IpaD serum IgA (**D**), and IpaH serum IgA (**E**) concentrations from healthy and sick individuals. Area under the curve (AUC), 95% confidence interval (CI), and *P* values are shown on the left; the pink dotted line indicates the optimal threshold value obtained from the corresponding AUC. Asterisks indicate statistically significant (*P* value < 0.05) differences in log-transformed pre-challenge antibody concentrations of healthy and sick individuals. *, *P* < 0.05; **, *P* < 0.01, as determined by unpaired *t*-test with Welch’s correction.

To identify immune markers associated with protective immunity, we applied PLSDA to compare antibody features in serum, lymphocyte supernatants, and fecal extracts collected before challenge (day 60) between individuals who remained healthy (disease index [DI] 0 or 1) and those who became sick (disease index 2 or 3) (Table S5) (15). Profiles from healthy and sick individuals were clearly distinct (Fig. 5A), and the top 10 discriminating features were all serum antibodies enriched in those who remained healthy (Fig. 5B). Putative protective thresholds were estimated by comparing pre-challenge serum antibody concentrations between healthy and sick groups using receiver operating characteristic (ROC) analysis (Fig. 5C through E). Antibodies with an area under the curve significantly greater than 0.5 included IpaB IgA (758.9 ng/mL) (Fig. 5C), IpaD IgA (270.9 ng/mL) (Fig. 5D), and IpaH IgA (481.5 ng/mL) (Fig. 5E). All were significantly higher immediately before challenge in individuals who remained healthy as compared to those who became sick (Fig. 5C through E, right panel).

## DISCUSSION

This article reports the breadth of systemic and mucosal humoral immunity elicited by WRSS1 vaccination and subsequent challenge with WT *S. sonnei* in Thai adults. The study uncovered antibody profiles in serum, ALS, and fecal extracts indicative of exposure or clinical protection in a *Shigella*-endemic population. It also revealed differences in antibody responses in the various compartments in terms of specificity and magnitude, as well as in relation to prior *Shigella* exposure.

Advancing *Shigella* vaccine candidates through clinical pathways currently relies on CHIM studies conducted in high-income countries (28). However, this approach fails to recapitulate vaccine performance in high-burden settings, where vaccines are needed. Several deemed promising in CHIM studies had low uptake and were poorly immunogenic in areas where *Shigella* is prevalent (15, 29). An understanding of natural and vaccine-induced immunity, and of immune components that can predict disease outcome, particularly in regions where *Shigella* is prevalent, is necessary to advance vaccine candidates. The Thai participants in the study had robust baseline humoral immunity to *Shigella* proteins; even those who became sick post-challenge had antibody levels that largely surpassed the threshold associated with protection against *S. flexneri* 2a in North American volunteers (Fig. S1) (18, 25, 30).

Herein, serum IgA specific to *Shigella* IpaB, IpaD, and IpaH emerged as distinctive features of non-infected individuals. This was unexpected, as protein-specific serum IgG had been singled out in US-based CHIM studies as a correlate of protection against *S. flexneri* 2a (8, 9, 31) and *S. sonnei* (10). Unlike IgG, which is abundant in serum, IgA participates in host defense at mucosal surfaces (32, 33). IgA in circulation is expected to be short-lived, and the robust levels observed in Thai adults likely reflect repeated exposure. Steady increases in serum IgA levels in young children from *Shigella*-endemic regions have been reported by our group and others (25, 34). It is unclear whether protein-specific IgA serves as a surrogate for an unknown protective component or exerts a direct antimicrobial function. A study conducted in a military base in Peru found an association between serum LPS-specific IgA-dependent functional activity and reduced risk of *Shigella* infection (35). In addition, our previous work demonstrating the concerted action of IgG and IgA in mediating bacteria killing *in vitro* (36) supports a potential mechanism through which IgA may contribute to protection.

ALS IgG and IgA responses post-vaccination and post-challenge were the most robust (one order of magnitude above those in serum or stool) and exhibited the broadest specificity, recognizing all antigens tested. ALS responses were identified as the most sensitive markers of *Shigella* exposure and shedding. Studies evaluating vaccine derivatives WRSs2 and WRSs3 reported a positive association between ALS responses and shedding (27). During the initial evaluation of WRSS1 vaccination and challenge, the duration of shedding was positively correlated with the magnitude of IgG- and IgA-ASC responses (15). Both ASC and ALS reflect the presence of orally primed, terminally differentiated B cells, which peak in circulation 7–10 days post-exposure and are considered markers of intestinal immunity (27). Together, these findings indicate that bacterial colonization, as reflected in prolonged shedding, primes the mucosal immunity and is necessary to elicit a systemic B-cell response against *Shigella*.

We observed substantial antibody reactivity against LPS from serotypes not represented in the vaccine or challenge strains (i.e., *S. flexneri* 2a, 3a, and 6), most prominently in lymphocyte supernatants and stool from naïve individuals post-challenge. These measurements were opportune, as the antigens were included in the multiplex assay. We interpret the heterologous LPS reactivity as antibody recognition of structurally similar epitopes across LPS molecules. B-cell immunity against these other serotypes from environmental exposure (37, 38) appears to have been boosted by *S. sonnei* vaccination and challenge.

Bactericidal ALS activity was muted despite elevated IgG and IgA levels. One possible explanation is that the ALS captures antibodies of relatively low affinity, originating from mucosally primed transient early plasmablasts before undergoing affinity maturation in lymphoid and mucosal sites (15, 39). Loss of functional activity due to sample handling and storage is also plausible. Future analysis of ALS antibody function, along with gut-homing B cells (e.g., α4β7), could provide insight into the types and antimicrobial capacities of antibodies relevant to mucosal immunity.

Local antibody responses in stool consisted primarily of IgA against both proteins and LPS, and robust bactericidal responses were detected post-vaccination and post-challenge. This finding is consistent with complement-mediated bactericidal responses in fecal extracts following WRSs2 and WRSs3 vaccination reported by our group (36). Although complement has traditionally been viewed as a systemic immune mechanism, increasing evidence indicates that complement proteins are produced locally and are functionally active within the human intestine. The synthesis of intestinal complement appears to be regulated by the microbiome (40, 41). In a mouse model of *Citrobacter rodentium* infection, lower complement C3 levels were associated with increased disease severity, and protection required complement-dependent neutrophil phagocytosis (42). These observations support a dual-action model in which the complement maintains intestinal homeostasis while preserving its capacity for microbial clearance through direct killing or by engaging antibodies and phagocytic cells. Complement proteins may also enter the infant gut via maternal milk; in earlier work, we demonstrated the presence of complement components C3 and C4 on the basolateral side of pediatric enteroids exposed to human breast milk (43). A combination of mucosal effector mechanisms involving antibodies, complement, and innate immune cells is anticipated for blocking *Shigella* infection in the human intestine.

Limitations of the study included the relatively small cohort size and the lack of active monitoring for other *Shigella* serotypes to which the participants could have been exposed during the study. In addition, the participants were adults, while the target population for *Shigella* protection is toddlers and young children (29).

In summary, the characterization of systemic and mucosal responses to *Shigella* antigens following *S. sonnei* vaccination and infection revealed ALS as broad markers of exposure and shedding and serum protein-specific IgA as putative correlates of protection in a region of high *Shigella* endemicity. Our findings provide further support for vaccines targeting conserved antigens, such as IpaB (44) and VirG (45), as promising candidates that can afford broad protection against multiple circulating strains.

## MATERIALS AND METHODS

### Clinical samples

Clinical samples examined in this study were collected from healthy adult volunteers aged 20–40 years participating in a Phase 1/2 clinical trial to assess the safety and efficacy of the *Shigella* WRSS1 vaccine (NCT01080716). The study design, screening criteria, methods, and vaccine safety and immunogenicity endpoints were described elsewhere (15, 24). Subjects were recruited from the Bangkok Metropolitan catchment area in Thailand. The clinical specimens (serum, lymphocyte supernatants, and fecal extracts) and corresponding time points are shown in Fig. 1.

Clinical signs and symptoms recorded in both the vaccine and challenge trials included dysentery, diarrhea, abdominal pain, nausea, headache, fatigue, rectal tenesmus, myalgia, malaise, stool consistency (loose, watery, with blood and mucus), frequency of stool, stool volume, temperature chart, severity of each individual based on the clinical symptoms, and early antibiotic intake (days).

DI was defined based on clinical symptoms, including gastrointestinal (GI) and systemic symptoms (Table S5). GI symptoms were defined as abdominal pain/cramps, nausea, vomiting, and anorexia. Systemic symptoms were defined as tenesmus, myalgia, joint pain, dizziness, malaise, headache, and fatigue. The DI scores of 0–3 were assigned as follows: a score of 0 was assigned to subjects who had no clinical symptoms, including no GI and systemic symptoms; a score of 1 was assigned to those who had no diarrhea, dysentery, and fever, but may have had other clinical symptoms; a score of 2 was assigned to those who had diarrhea and/or dysentery accompanied by fever, 1 or 2 bloody stools, or 1 or 2 loose stools; and a score of 3 was assigned to those who had diarrhea and/or dysentery accompanied by fever, had 3–10 bloody stools, or 3–10 loose stools.

### Antigen-specific IgG and IgA binding analysis

Preparation of serum, lymphocyte supernatants, and fecal extracts for antibody binding assays was described in detail previously (15). IgG and IgA specific for *Shigella* antigens were measured in serum, fecal extract, and lymphocyte supernatants using a qualified MesoScale Discovery (MSD, Rockville, MD) ECLIA (25). Briefly, 96-well MSD plates spotted with the *Shigella* antigens IpaB, IpaC, IpaD, IpaH, VirG, and LPS specific to *S. flexneri* (2a, 3a, and 6) and *S. sonnei* were blocked with 150 μL of 5% BSA in PBS for 1 h, followed by the addition of diluted samples and incubation for 90 min with shaking. In-house standards were used to calculate antibody titers. The IgG serum standard was previously described (25). An IgA standard was generated by pooling equal volumes of high-titer sera from this cohort. *Shigella* antigen-specific IgG and IgA concentrations in the standards were determined by the Zollinger method, as previously described (25, 46). The standards, positive control (1:5,000 for IgG and 1:200 for IgA), and negative control (human serum minus IgA/IgM/IgG; Sigma-Aldrich, St. Louis, MO) were included on each plate. All incubations were at room temperature with shaking at 700 rpm. Between incubations, the plates were washed three times with 300 μL of PBS containing 0.05% Tween 20. For antibody detection, plates were incubated for 1 h with 50 μL of biotinylated-SP (long spacer) AffiniPure goat anti-human IgA (α-chain specific) or IgG (Fcγ fragment specific; Jackson ImmunoResearch Laboratories, Inc., West Grove, PA) followed by SULFO-TAG Streptavidin, for 1 h. The MSD GOLD Read Buffer A was added, and the plate was read in an MSD QuickPlex SQ 120 reader within 20 min. The data were analyzed using MSD Discovery Workbench (version 4.0, MSD). Blanks were subtracted from ECL values of test samples, and antibody concentrations were calculated by backfitting in the standard 4PL curves.

### Functional antibody analysis

*S. sonnei* BA and OPKA were measured as previously described (11, 47). Briefly, for BA, heat-inactivated (56°C for 30 min) samples were serially diluted in assay buffer (0.1% gelatin [Sigma-Aldrich] in HBSS with Ca^2+^ and Mg^2+^ [Invitrogen, Carlsbad, CA]) and mixed with *S. sonnei* strain Moseley (target = 100 CFU per well) and 12.5% (final concentration) baby rabbit complement (BRC; Pel-Freez Biologicals, Rogers, AR). The plates containing samples, target bacteria, and BRC were briefly mixed by shaking at 700 rpm for 10–15 s and incubated at 37°C for 2 h. For OPKA, heat-inactivated, serially diluted serum was incubated with *S. sonnei* Moseley for 15 min at 37°C with shaking, then incubated with differentiated HL-60 cells and BRC (10% final concentration) for 45 min at 37°C, 5% CO_2_. Following opsonization, 10 μL of the reaction mixture was spotted onto square Luria-Bertani agar plates (BD Diagnostics Systems, Sparks, MD) and incubated at 26°C for 16–18 h. Titers were calculated as the killing index, defined as the reciprocal of the sample dilution required to achieve 50% bacterial killing (11). A human internal reference serum, Korea PnQC19, was included as a positive control in each run.

### Statistical analysis

Antibody concentrations (ng/mL) and lower limit of quantification (LLOQ) for each antigen were determined from the individual standard curves as previously described (25). Concentrations below this limit were assigned an LLOQ value. Antibody responses were represented as GMFRs. Responders were defined as those with a ≥4-fold increase in antibody levels over baseline (day 0 or 60, respectively) after vaccination or challenge. Heat maps were created using GraphPad Prism (version 10; GraphPad, San Diego, CA). PLSDA was performed using the mixOmics R package (48). ROC analysis was performed using pre-challenge antibody concentrations from individuals who remained healthy (DI = 0 or 1) vs those who were sick (DI = 2 or 3). Optimal antibody concentration thresholds were estimated using the nearest-to (0,1) method (49, 50), with sensitivity and specificity of ≥75%. Comparison of antibody levels in healthy and sick individuals was performed by an unpaired *t*-test with Welch’s correction. *P* values of<0.05 were considered statistically significant. All statistical analyses were performed using MSD Discovery Workbench, GraphPad Prism (version 10, GraphPad), or R (version 4.5.2).

## Data Availability

All raw IgG and IgA concentrations calculated from serum, lymphocyte supernatants, and fecal samples are provided in Table S6.

## SUPPLEMENTAL MATERIAL

Supplemental Material msphere.00269-26-s0001.docx Tables S1-S5; Figure S1.

Legends msphere.00269-26-s0002.docx Supplemental legends.

Table S6 msphere.00269-26-s0003.xlsx Raw antibody concentrations (pg/mL) in serum, lymphocyte supernatants, and fecal supernatants.

ASM does not own the copyrights to Supplemental Material that may be linked to, or accessed through, an article. The authors have granted ASM a non-exclusive, world-wide license to publish the Supplemental Material files. Please contact the corresponding author directly for reuse.
