# Supplementary material for: Protective immunity against Shigella sonnei in vaccinated and challenged adult Thai volunteers
Source: mSphere. 2026 Jul 30;11(8):e00269-26. doi: 10.1128/msphere.00269-26 (PMC13508790; doi:10.1128/msphere.00269-26)
Supplement: Supplementary file 1 [file msphere.00269-26-s0001.docx]

| **Table S1. Geometric Mean Fold Rise (GMFR) of *Shigella* antibodies in serum post-vaccination and challenge** | | | | | | | | |
| --- | --- | --- | --- | --- | --- | --- | --- | --- |
|  | **Post-vaccination** | | | | **Post-challenge** | | | |
|  | **Day 14** | | **Day 28** | | **Day 74** | | **Day 88** | |
|  | Placebo  controls  (n=6) | Vaccinees  (n=13) | Placebo  Controls  (n=6) | Vaccinees  (n=13) | Naïve  controls  (n=10) | Vaccinees  (n=10) | Naïve  controls  (n=10) | Vaccinees  (n=10) |
|  | GMFR  95% CI | GMFR  95% CI | GMFR  95% CI | GMFR  95% CI | GMFR  95% CI | GMFR  95% CI | GMFR  95% CI | GMFR  95% CI |
| **IgG** | | | | | | | | |
| IpaB | 1.2  (0.7, 1.7) | 3.2  (1.8, 4.7) | 1.2  (0.5, 1.9) | **4.6**  (0.7, 8.7) | **5.6**  (2.1, 9.1) | **4.1**  (1.6, 6.5) | **6.2**  (0.6, 12.2) | 3.7  (0.3, 7.3) |
| IpaC | 1.4  (0.3, 2.5) | **4.3**  (2.7, 5.9) | 1.5  (0.1, 2.9) | **4.3**  (2.3, 6.3) | **6.2**  (2.2, 10.3) | 3.9  (3.1, 4.7) | **5.2**  (1.8, 8.7) | 3.7  (2.8, 4.6) |
| IpaD | 1.1  (0.7, 1.5) | 3.4  (2.6, 4.2) | 1.2  (0.6, 1.9) | 3.5  (2.6, 4.4) | **5.1**  (2.6, 7.7) | **4.1**  (3.1, 5.1) | **4.9**  (2.4, 7.5) | 3.9  (2.8, 5.1) |
| IpaH | 1.3  (0.1, 2.6) | 3.2  (2.4, 4.0) | 1.4  (-0.1, 2.9) | 3.3  (2.3, 4.4) | **7.1**  (2.4, 12.0) | 3.9  (2.9, 5.0) | **6.6**  (1.7, 11.8) | 3.7  (2.6, 4.8) |
| VirG | 1.4  (0.3, 2.5) | 2.6  (1.8, 3.5) | 1.3  (0.3, 2.3) | 2.6  (1.8, 3.5) | 3.9  (2.5, 5.4) | 3.8  (2.8, 4.7) | **4.4**  (2.8, 6.1) | 3.0  (1.9, 4.2) |
| *Ss*^1^ LPS | 1.3  (0.8, 1.8) | **4.0**  (-0.02,8.3) | 1.3  (0.4, 2.3) | **4.9**  (-0.8, 10.8) | **11.9**  (-0.2, 25.5) | **7.9**  (0.5, 15.8) | **11.3**  (0.5, 23.3) | **6.6**  (0.4, 13.3) |
| **IgA** | | | | | | | | |
| IpaB | 0.9  (0.2, 1.6) | 3.3  (1.1, 5.5) | 1.6  (0.3, 2.8) | 2.7  (1.6, 3.8) | **13.2**  (1.4,26.4) | **4.4**  (-1.5, 10.6) | 3.3  (0.9, 5.7) | 2.3  (-0.3, 5.0) |
| IpaC | 1.0  (0.2, 1.8) | 3.3  (1.8, 4.8) | 1.6  (0.4, 2.8) | 3.2  (1.8, .6) | **4.2**  (-0.6, 9.3) | 1.8  (0.8, 2.7) | 2.4  (0.7, 4.2) | 1.4  (0.8, 2.0) |
| IpaD | 0.9  (0.2, 1.6) | **4.1**  (1.6, 6.6) | 1.5  (0.4, 2.6) | 3.5  (2.0, 5.0) | **4.7**  (0.2, 9.3) | 1.9  (0.7, 3.0) | 2.4  (1.0, 3.9) | 1.4  (0.8, 2.1) |
| IpaH | 1.0  (0.2, 1.8) | 2.9  (1.4, 4.5) | 1.5  (0.4, 2.7) | 2.8  (1.7, 4.0) | 2.6  (0.4, 4.8) | 2.4  (0.3, 4.6) | 1.7  (0.8, 2.5) | 2.0  (0.1, 4.0) |
| VirG | 1.0  (0.2, 1.9) | 2.6  (1.8, 3.5) | 1.6  (0.4, 2.8) | 2.6  (1.8, 3.5) | **4.2**  (1.1, 7.3) | 3.1  (0.2, 6.1) | 2.2  (0.8, 3.6) | 1.9  (0.5, 3.3) |
| *Ss*^1^ LPS | 0.9  (0.2, 1.5) | 2.4  (1.8, 3.0) | 1.4  (0.4, 2.5) | 2.4  (1.8, 3.0) | **22**  (-1.4, 50.9) | **21.2**  (-0.4, 47.6) | **5.2**  (0.2, 10.4) | **5.1**  (1.0, 9.5) |
| **Functional** | | | | | | | | |
| SBA | 1.1  (0.8, 1.4) | **4.9**  (-0.6,10.7) | 0.8  (0.5, 1.1) | **4.4**  (-0.5, 9.6) | **11.4**  (-2.2, 26.8) | **16.9**  (0.8, 35.6) | **6.6**  (-1.5, 15.3) | **12.0**  (-0.8, 26.5) |
| OPKA | 1.0  (0.6, 1.4) | 3.2  (0.7, 5.8) | 1.4  (0.3, 2.5) | 1.7  (0.7, 2.7) | **9.2**  (-1.0, 20.5) | **6.4**  (0.7, 12.5) | **7.6**  (-0.4, 16.3) | **4.3**  (0.7, 8.1) |
| ^1^*Ss*, *S. sonnei* | | | | | | | | |

| **Table S2. GMFR of *Shigella* antibodies in lymphocyte supernatants post-vaccination and challenge** | | | | |
| --- | --- | --- | --- | --- |
|  | **Post-vaccination** | | **Post-challenge** | |
|  | **Day 9** | | **Day 69** | |
|  | Placebo  Controls  (n=6) | Vaccinees  (n=13) | Naïve  Controls  (n=10) | Vaccinees  (n=10) |
|  | GMFR  95% CI | GMFR  95% CI | GMFR  95% CI | GMFR  95% CI |
| **IgG** | | | | |
| IpaB | 1.4  (0.4, 2.5) | **35.0**  (-1.1, 84.2) | **120.9**  (16.0, 320.7) | **79.9**  (2.2, 216.9) |
| IpaC | 1.7  (0.3, 3.1) | **31.6**  (-3.6, 79.6) | **88.0**  (5.4, 235.3) | **85.7**  (2.7, 235.8) |
| IpaD | 1.2  (0.1, 2.3) | **49.3**  (-6.8, 139.3) | **101.0**  (7.6, 275.2) | **109.0**  (-4.3, 356.5) |
| IpaH | 1.2  (0.3, 2.1) | **35.8**  (-10.1, 105.3) | **109.8**  (6.7, 312.3) | **89.2**  (-1.6, 263.7) |
| VirG | 1.3  (0.2, 2.3) | 2.4  (0.4, 4.5) | **91.4**  (8.0, 239.3) | **73.5**  (-8.3, 228.1) |
| *Ss*^1^ LPS | 0.8  (0.4, 1.2) | **64.1**  (-10.1, 199.3) | **594.7**  (107.9, 2220.7) | **303.7**  (29.7, 1156.7) |
| **IgA** | | | | |
| IpaB | 1  (1, 1) | **39.2**  (1.4, 90.9) | **52.7**  (-9.0, 156.4) | **52.7**  (-5.7, 147.1) |
| IpaC | 1  (1, 1) | **20.1**  (-0.9, 45.6) | **39.3**  (13.0, 72.0) | **61.6**  (-5.2, 175.7) |
| IpaD | 1  (1, 1) | **22.5**  (0.3, 49.8) | **30.1**  (-5.3, 78.8) | **49.6**  (-8.0, 143.3) |
| IpaH | 1.0  (0.9, 1.1) | **13.7**  (-1.6, 31.5) | **25.0**  (5.4, 48.2) | **31.0**  (-5.9, 82.5) |
| VirG | 1  (1, 1) | 2.6  (0.3, 4.9) | **79.5**  (18.4, 172.0) | **41.8**  (-4.6, 110.8) |
| *Ss*^1^ LPS | 0.9  (0.5, 1.2) | **51.6**  (-1.3, 133.0) | **177.0**  (59.3, 381.4) | **71.9**  (-8.6, 223.4) |
| **Functional** | | | | |
| *Ss*^1^ ALS BA | 0.9  (0.7, 1.1) | 1.2  (1.1, 1.4) | 2.5  (1.4, 3.6) | 1.5  (1.1, 1.8) |
| *Ss*^1^ ALS OPKA | 1.2  (0.6, 1.8) | 1.8  (-0.1, 3.8) | 1.3  (0.8, 1.8) | 0.6  (0.3, 0.9) |
| ^1^*Ss*, *S. sonnei* | | | | |

| **Table S3a. GMFR of *Shigella* antibodies in fecal supernatants post-vaccination** | | | | | | | | |
| --- | --- | --- | --- | --- | --- | --- | --- | --- |
|  | **Day 5** | | **Day 7** | | **Day 14** | | **Day 28** | |
|  | Placebo  controls  (n=6) | Vaccinees  (n=13) | Placebo  controls  (n=6) | Vaccinees  (n=13) | Placebo  controls  (n=6) | Vaccinees  (n=13) | Placebo  controls  (n=6) | Vaccinees  (n=13) |
|  | GMFR  95% CI | GMFR  95% CI | GMFR  95% CI | GMFR  95% CI | GMFR  95% CI | GMFR  95% CI | GMFR  95% CI | GMFR  95% CI |
| **IgG** | | | | | | | | |
| IpaB | 1.3  (0.5, 2.2) | 1.5  (-0.1, 3.2) | 1.4  (0.3, 2.5) | 2.5  (0.4, 4.6) | 1.0  (1, 1) | 1.0  (0.5, 1.4) | 1.0  (1, 1) | 3.3  (-1.1, 7.9) |
| IpaC | 1.0  (1, 1) | 0.9  (0.7, 1.2) | 1.0  (1, 1) | 0.9  (0.7, 1.2) | 1.0  (1, 1) | 0.9  (0.6, 1.1) | 1.0  (1, 1) | 1.9  (-0.3, 4.0) |
| IpaD | 1.0  (1, 1) | 0.9  (0.6, 1.2) | 1.0  (1, 1) | 1.2  (0.6, 1.8) | 1.0  (1, 1) | 0.9  (0.6, 1.1) | 1.0  (1, 1) | 3.5  (-2.0, 9.3) |
| IpaH | 1.0  (1.0, 1.0) | 0.8  (0.6, 1.1) | 1.0  (1, 1) | 0.8  (0.6, 1.0) | 1.0  (1, 1) | 0.8  (0.6, 1.1) | 1.0  (1, 1) | 2.2  (-0.7, 5.3) |
| VirG | 1.1  (0.7, 1.6) | 1.3  (0.2, 2.5) | 1.2  (0.7, 1.7) | 1.5  (0.4, 2.7) | 1.0  (0.9, 1.0) | 1.3  (0.2, 2.4) | 1.1  (0.7, 1.5) | **4.6**  (-2.7, 12.3) |
| *Ss*^1^ LPS | 1.4  (0.3, 2.6) | 0.7  (0.4, 1.0) | 1.3  (0.6, 2.1) | 1.5  (0.45, 2.5) | 1.6  (-0.3, 3.6) | 3.2  (0.8, 5.6) | 1.3  (0.2, 2.4) | **7.0**  (-3.5, 18.6) |
| **IgA** | | | | | | | | |
| IpaB | 1.0  (0.2, 1.7) | 1.0  (0.4, 1.6) | 1.3  (-0.3, 3.0) | **4.1**  (-1.0, 9.4) | 1.3  (0.1, 2.4) | **4.8**  (1.1, 8.6) | **6.2**  (-6.8, 21.0) | **4.9**  (-0.1, 10.0) |
| IpaC | 0.9  (0.4, 1.5) | 1.0  (0.4, 1.5) | 0.8  (0.2, 1.5) | 2.1  (0.7, 3.5) | 0.7  (0.2, 1.1) | 2.0  (0.7, 3.3) | **6.5**  (-7.8, 23.0) | 3.0  (-0.1, 6.1) |
| IpaD | 1.7  (0.3, 3.0) | 1.0  (0.4, 1.7) | 1.9  (-0.5, 4.3) | 2.5  (0.6, 4.5) | 2.2  (-1.9, 6.5) | **6.7**  (-4.3, 19.0) | **9.5**  (-11.9, 36.1) | 2.3  (0.3, 4.4) |
| IpaH | 1.0  (0.3, 1.7) | 0.8  (0.3, 1.4) | 1.0  (0.2, 1.9) | 2.3  (0.7, 4.0) | 0.8  (-0.3, 1.9) | 2.2  (0.4, 4.0) | **11.3**  (-14.0, 44.0) | 2.8  (-0.1, 5.7) |
| VirG | 0.9  (0.2, 1.5) | 0.7  (0.3, 1.0) | 1.0  (0.2, 1.8) | 3.0  (-0.1, 6.3) | 2.9  (-1.4, 7.4) | **7.0**  (-2.3, 17.4) | **7.6**  (-8.5, 26.5) | 2.9  (1.1, 4.6) |
| *Ss*^1^ LPS | 2.5  (-0.9, 6.0) | 0.8  (0.4, 1.2) | 1.5  (-0.1, 3.1) | **4.3**  (-1.5, 10.4) | 1.1  (-0.1, 2.2) | **19.5**  (4.1, 37.1) | **6.3**  (-7.6, 22.3) | **5.0**  (1.7, 8.4) |
| **Functional** | | | | | | | | |
| Fecal BA | 1.0  (1, 1) | 0.9  (0.6, 1.1) | 1.0  (1, 1) | 1.4  (0.5, 2.2) | 1.5  (0.2, 2.8) | **57.4**  (-2.5, 154) | 1.0  (1, 1) | 1.0  (0.6, 1.4) |
| Fecal  OPKA | 1.0  (1, 1) | 0.9  (0.8, 1.0) | 1.0  (1, 1) | 1.1  (0.7, 1.5) | 1.9  (0.4, 3.4) | **11.3**  (-1.5, 25.6) | 1.9  (-0.4, 4.2) | 2.7  (-0.4, 5.8) |
| ^1^*Ss*, *S. sonnei* | | | | | | | | |

| **Table S3b. GMFR of *Shigella* antibodies in fecal supernatants post-challenge** | | | | | | |  | |
| --- | --- | --- | --- | --- | --- | --- | --- | --- |
|  | **Day 65** | | **Day 67** | | **Day 74** | | **Day 88** | |
|  | Naïve  Controls  (n=10) | Vaccinees  (n=10) | Naïve  controls  (n=10) | Vaccinees  (n=10) | Naïve  controls  (n=10) | Vaccinees  (n=10) | Naïve  controls  (n=10) | Vaccinees  (n=10) |
|  | GMFR  95% CI | GMFR  95% CI | GMFR  95% CI | GMFR  95% CI | GMFR  95% CI | GMFR  95% CI | GMFR  95% CI | GMFR  95% CI |
| **IgG** | | | | | | |  | |
| IpaB | 0.8  (0.6, 1.1) | 2.2  (-1.1, 5.7) | 2.0  (-0.2, 4.2) | 1.6  (0.4, 2.7) | 2.3  (0.1, 4.5) | **4.0**  (-3.3, 11.7) | 1.0  (0.6, 1.4) | 3.6  (-1.2, 8.6) |
| IpaC | 0.9  (0.7, 1.1) | 1.1  (0.5, 1.7) | 0.9  (0.7, 1.1) | 1.0  (0.6, 1.4) | 0.9  (0.7, 1.1) | 0.9  (0.7, 1.1) | 1.7  (-0.2, 3.7) | 1.1  (0.6, 1.7) |
| IpaD | 1.1  (0.5, 1.7) | 1.4  (0.2, 2.5) | 1.7  (0.5, 2.9) | 1.5  (0.1, 2.8) | 1.3  (0.8, 1.7) | 0.8  (0.6, 1.1) | 2.3  (-0.1, 4.8) | 1.2  (0.5, 1.8) |
| IpaH | 2.2  (-0.6, 5.1) | 1.7  (0.5, 2.8) | 1.0  (0.8, 1.2) | 1.3  (0.3, 2.3) | 0.9  (0.8, 1.1) | 0.9  (0.7, 1.1) | 1.6  (0.2, 3.1) | 1.0  (0.7, 1.3) |
| VirG | 0.7  (0.4, 1.0) | **8.1**  (-8.0, 26.9) | 2.2  (0.5, 4.0) | 1.7  (0.6, 2.8) | 2.0  (0.8, 3.2) | 0.8  (0.5, 1.1) | 1.4  (0.2, 2.6) | 1.5  (-0.1, 3.2) |
| *Ss*^1^ LPS | 0.5  (0.1, 1.0) | **5.5**  (-2.7, 14.5) | 1.5  (0.7, 2.2) | **10.2**  (-1.1, 22.6) | **6.0**  (-2.7, 15.4) | **22.3**  (-8.8, 64.0) | 2.0  (-0.3, 4.4) | **19.8**  (-3.5, 48.6) |
| **IgA** | | | | | | |  | |
| IpaB | **10.1**  (-1.1, 22.6) | **9.0**  (-6.4, 27.0) | **22.2**  (5.2, 41.8) | **13.4**  (-5.7, 36.3) | **10.4**  (-1.6, 23.9) | **8.4**  (-4.9, 23.5) | **15.6**  (-6.9, 43.6) | **9.1**  (-6.3, 27.0) |
| IpaC | 2.1  (0.4, 3.9) | **5.6**  (-3.2, 15.2) | 3.1  (0.9, 5.3) | **6.5**  (-3.6, 17.7) | 1.5  (0.4, 2.6) | 3.9  (-3.1, 11.4) | 1.7  (0.1, 3.4) | **7.2**  (-4.9, 20.8) |
| IpaD | 2.3  (0.9, 3.7) | **8.7**  (-4.9, 24.2) | 3.4  (1.0, 5.9) | **9.0**  (-4.2, 23.9) | 1.9  (0.5, 3.3) | **6.0**  (-4.6, 17.7) | 2.3  (0.4, 4.2) | **6.6**  (-2.6, 16.7) |
| IpaH | **5.8**  (-0.4, 12.4) | **10.7**  (-9.2, 34.9) | **7.3**  (-1.8, 17.2) | **9.8**  (-5.6, 27.7) | 2.1  (0.2, 4.1) | 3.8  (-2.5, 10.4) | 2.6  (0.2, 5.1) | **8.3**  (-6.0, 24.7) |
| VirG | **4.2**  (0.6, 8.0) | **11.7**  (-5.5, 31.9) | **12.6**  (1.8, 24.6) | **16.8**  (-5.6, 44.4) | 3.6  (0.7, 6.7) | **14.0**  (-6.4, 38.9) | **5.5**  (0.2, 11.1) | **11.4**  (-7.0, 33.3) |
| *Ss*^1^ LPS | 1.6  (0.4, 2.8) | **4.2**  (-1.5, 10.4) | **8.4**  (2.5, 14.6) | **17.1**  (-8.8, 50.3) | **9.9**  (-2.4, 23.7) | **20.7**  (-9.7, 61.3) | 3.6  (0.5, 6.7) | **16.7**  (-10.8, 52.8) |
| **Functional** | | | | | | |  | |
| SBA | **8.6**  (-2.5, 21.1) | 2.0  (0.0, 4.0) | **17.2**  (4.7, 31.3) | **8.3**  (1.3, 15.9) | **228.6**  (76.2, 513.0) | **21.9**  (-3.7, 54.4) | **34.8**  (-1.4, 84.2) | **11.9**  (-7.6, 35.5) |
| OPKA | 3.2  (0.5, 5.9) | 3.6  (-0.4, 7.8) | **10.8**  (1.0, 21.6) | **8.8**  (1.9, 16.2) | **61.7**  (14.8, 127.9) | **7.3**  (-4.0, 19.9) | **12.5**  (2.7, 23.3) | 2.1  (1.0, 3.2) |
| ^1^*Ss*, *S. sonnei* | | | | | | | | |

| **Table S4a. GMFR of *Shigella* antibodies in serum post-vaccination and challenge** | | | | | | | | | |
| --- | --- | --- | --- | --- | --- | --- | --- | --- | --- |
|  | **Post-vaccination** | | | | | **Post-challenge** | | | |
|  | **Day 14** | | | **Day 28** | | **Day 74** | | **Day 88** | |
|  | Placebo  Controls  (n=6) | Vaccinees  (n=13) | Placebo  controls  (n=6) | | Vaccinees  (n=13) | Naïve  controls  (n=10) | Vaccinees  (n=10) | Naïve  controls  (n=10) | Vaccinees  (n=10) |
|  | GMFR  95% CI | GMFR  95% CI | GMFR  95% CI | | GMFR  95% CI | GMFR  95% CI | GMFR  95% CI | GMFR  95% CI | GMFR  95% CI |
| **IgG** | | | | | | | | | |
| *Sf*2a LPS | 1.10  (0.8, 1.5) | 2.6  (0.5, 4.8) | 1  (0.7, 1.4) | | **4.9**  (-1.5, 11.7) | **6.3**  (1.0, 11.9) | 2.6  (1.7, 3.5) | **4.7**  (0.4, 9.3) | 1.9  (1.4, 2.4) |
| *Sf*3a LPS | 1.10  (0.7, 1.6) | 2.6  (0.3, 4.9) | 1.3  (0.6, 1.9) | | 3.2  (-0.3, 6.9) | **7**  (-2.6, 17.4) | 2.1  (1.7, 2.5) | 2.8  (0.6, 5.1) | 2.6  (1.4, 3.8) |
| *Sf*6 LPS | 1.10  (0.7, 1.7) | 1.4  (1.1, 1.7) | 1.4  (0.5, 2.3) | | 1.6  (1.2, 1.9) | **4.5**  (1.6, 7.4) | 2  (1.5, 2.4) | 3.8  (1.4, 6.2) | 2.2  (1.2, 3.2) |
| **IgA** | | | | | | | | | |
| *Sf*2a LPS | 1.10  (0.3, 1.9) | 2.2  (1.6, 2.8) | 1.5  (0.5, 2.6) | | 2.3  (1.8, 2.8) | 1.4  (0.8, 2.0) | 1.3  (0.9, 1.6) | 1.3  (0.7, 2.0) | 1.1  (0.8, 1.4) |
| *Sf*3a LPS | 1.00  (0.2, 1.8) | 2.2  (1.6, 2.9) | 1.5  (0.4, 2.6) | | 2.3  (1.8, 2.8) | 1.2  (0.8, 1.6) | 1.1  (0.8, 1.4) | 1.3  (0.7, 1.9) | 1.0  (0.8, 1.3) |
| *Sf*6 LPS | 0.90  (0.1, 1.7) | **8.2**  (2.1, 14.7) | 1.4  (0.4, 2.5) | | **4.6**  (2.0, 7.3) | 2.4  (-0.3, 5.3) | 1  (0.7, 1.2) | 1.3  (0.8, 1.7) | 1.0  (0.7, 1.3) |
| *Sf*2a, *S. flexneri* 2a; *Sf*3a, *S. flexneri* 3a; *Sf*6, S. *flexneri* 6. | | | | | | | | | |

| **Table S4b. GMFR of *Shigella* antibodies in lymphocyte supernatants post-vaccination and challenge** | | | | |
| --- | --- | --- | --- | --- |
|  | **Post-vaccination** | | **Post-challenge** | |
|  | **Day 9** | | **Day 69** | |
|  | Placebo  Controls  (n=6) | Vaccinees  (n=13) | Naïve  Controls  (n=10) | Vaccinees  (n=10) |
|  | GMFR  95% CI | GMFR  95% CI | GMFR  95% CI | GMFR  95% CI |
| **IgG** | | | | |
| *Sf*2a LPS | 1.2  (0.4, 2.0) | **14.4**  (-2.8, 34.6) | **40.8**  (-0.7, 99.5) | **25.9**  (-4.3, 65.7) |
| *Sf*3a LPS | 1.0  (0.2, 1.9) | **4.2**  (-0.1, 8.6) | **15.5**  (4.0, 28.2) | **6.9**  (-1.5, 15.9) |
| *Sf*6 LPS | 0.8  (0.2, 1.5) | **8.6**  (-1.6, 19.7) | **134.6**  (6.0, 419.1) | **29.6**  (-4.3, 75.7) |
| **IgA** | | | | |
| *Sf*2a LPS | 1.4  (0.4, 2.4) | 3.2  (1.1, 5.4) | **9.1**  (3.2, 15.4) | **9.1**  (-1.7, 20.9) |
| *Sf*3a LPS | 1.0  (0.9, 1.2) | 1.7  (0.5, 2.8) | 1.7  (0.3, 3.1) | 1.7  (0.2, 3.2) |
| *Sf*6 LPS | 1.3  (0.6, 2.0) | **6.3**  (-0.2, 13.3) | **56.6**  (-5.2, 158.5) | **5.7**  (-3.5, 15.7) |
| *Sf*2a, *S. flexneri* 2a; *Sf*3a, *S. flexneri* 3a; *Sf*6, S. *flexneri* 6. | | | | |

| **Table S4c. GMFR of *Shigella* antibodies post-vaccination in fecal supernatants** | | | | | | |  | |
| --- | --- | --- | --- | --- | --- | --- | --- | --- |
|  | **Day 5** | | **Day 7** | | **Day 14** | | **Day 28** | |
|  | Placebo  controls  (n=6) | Vaccinees  (n=13) | Placebo  controls  (n=6) | Vaccinees  (n=13) | Placebo  controls  (n=6) | Vaccinees  (n=13) | Placebo  controls  (n=6) | Vaccinees  (n=13) |
|  | GMFR  95% CI | GMFR  95% CI | GMFR  95% CI | GMFR  95% CI | GMFR  95% CI | GMFR  95% CI | GMFR  95% CI | GMFR  95% CI |
| **IgG** | | | | | | | | |
| *Sf*2a LPS | 1.1  (0.8, 1.5) | 0.8  (0.6, 1.0) | 1.3  (0.7, 2.0) | 1.5  (0.6, 2.6) | 0.9  (0.8, 1.1) | 0.8  (0.6, 1.0) | 1.0  (0.6, 1.3) | 1.9  (0.1, 3.8) |
| *Sf*3a LPS | 0.9  (0.6, 1.2) | 1.1  (0.6, 1.6) | 1.8  (-0.2, 3.9) | 1.4  (0.7, 2.1) | 0.8  (0.6, 1.1) | 1.2  (0.3, 2.1) | 1.5  (-0.2, 3.2) | **12.6**  (-10.5, 41.7) |
| *Sf*6 LPS | 0.9  (0.6, 1.2) | 0.9  (0.7, 1.1) | 1.0  (0.8, 1.3) | 1.3  (0.7, 1.9) | 0.9  (0.6, 1.2) | 0.8  (0.6, 1.0) | 1.2  (0.3, 2.1) | 2.7  (0.4, 5.0) |
| **IgA** | | | | | | | | |
| *Sf*2a LPS | 1.4  (0.1, 2.7) | 1.9  (-0.1, 4.0) | 1.8  (-0.3, 4.0) | **4.6**  (0.2, 9.3) | 1.0  (-0.1, 2.1) | **9.4**  (-2.8, 23.1) | **7.0**  (-8.1, 24.6) | **4.5**  (0.6, 8.4) |
| *Sf*3a LPS | 1.3  (-0.1, 2.9) | 1.4  (0.4, 2.5) | 1.5  (-0.3, 3.2) | **5.3**  (-0.3, 11.1) | 1.5  (-1.0, 4.0) | 3.3  (0.6, 6.1) | **7.5**  (-9.1, 27.2) | **5.4**  (-1.7, 13.1) |
| *Sf*6 LPS | 1.2  (-0.1, 2.6) | 1.2  (0.3, 2.2) | 1.5  (-0.5, 3.5) | **5.3**  (0.3, 10.5) | 1.3  (-0.7, 3.3) | **5.5**  (1.3, 9.8) | **16.7**  (20.1, 70.4) | **5.2**  (-0.9, 11.7) |
| *Sf*2a, *S. flexneri* 2a; *Sf*3a, *S. flexneri* 3a; *Sf*6, S. *flexneri* 6. | | | | | | | | |

| **Table S4d. GMFR of *Shigella* antibodies post-challenge in fecal supernatants** | | | | | | |  | |
| --- | --- | --- | --- | --- | --- | --- | --- | --- |
|  | **Day 65** | | **Day 67** | | **Day 74** | | **Day 88** | |
|  | Naïve  controls  (n=10) | Vaccinees  (n=10) | Naïve  controls  (n=10) | Vaccinees  (n=10) | Naïve  controls  (n=10) | Vaccinees  (n=10) | Naïve  controls  (n=10) | Vaccinees  (n=10) |
|  | GMFR  95% CI | GMFR  95% CI | GMFR  95% CI | GMFR  95% CI | GMFR  95% CI | GMFR  95% CI | GMFR  95% CI | GMFR  95% CI |
| **IgG** | | | | | | | | |
| *Sf*2a LPS | 0.5  (0.3, 0.8) | 1.5  (-0.3, 3.3) | 1.1  (0.6, 1.6) | 0.9  (0.5, 1.2) | 1.1  (0.5, 1.6) | 1.0  (0.4, 1.7) | 1.7  (-0.4, 3.9) | 1.0  (0.5, 1.4) |
| *Sf*3a LPS | 0.5  (0.2, 0.7) | **5.0**  (-4.7, 15.8) | 1.4  (0.5, 2.4) | 1.1  (0.6, 1.6) | 1.0  (0.8, 1.2) | 1.2  (0.0, 2.4) | 1.9  (-0.1, 3.8) | 1.3  (0.5, 2.1) |
| *Sf*6 LPS | 0.5  (0.2, 0.8) | 3.8  (-3.0, 10.9) | 1.2  (0.5, 2.0) | 1.2  (0.4, 2.0) | 0.9  (0.7, 1.1) | 1.8  (0.0, 3.5) | 1.6  (-0.1, 3.3) | 2.8  (-0.7, 6.3) |
| **IgA** | | | | | | | | |
| *Sf*2a LPS | **12.1**  (0.3, 25.3) | **6.5**  (-0.9, 14.5) | **33.9**  (6.0, 69.0) | **8.2**  (-0.0, 17.0) | **8.3**  (-4.4, 22.7) | 2.3  (0.1, 4.6) | **10.5**  (-4.7, 28.1) | 3.4  (-0.7, 7.7) |
| *Sf*3a LPS | **8.0**  (0.4, 16.1) | **7.1**  (-0.3, 15.1) | **23.8**  (4.7, 46.3) | **9.6**  (0.4, 19.6) | **8.6**  (-5.6, 24.9) | 2.5  (0.2, 4.8) | **6.6**  (-2.7, 16.7) | **4.0**  (-0.7, 9.0) |
| *Sf*6 LPS | **6.2**  (0.6, 12.1) | **8.0**  (-3.1, 20.5) | **16.9**  (4.6, 30.5) | **9.9**  (-3.2, 24.7) | **4.6**  (-1.5, 11.1) | **5.2**  (0.0, 10.7) | **7.5**  (-5.4, 22.1) | **4.8**  (-1.6, 11.7) |
| *Sf*2a, *S. flexneri* 2a; *Sf*3a, *S. flexneri* 3a; *Sf*6, S. *flexneri* 6. | | | | | | | | |

| Table S5. Disease severity scores and clinical symptoms in Thai adults WRSS1 vaccination and subsequent *S. sonnei* 53G challenge | | | | | | | | | |
| --- | --- | --- | --- | --- | --- | --- | --- | --- | --- |
|  | **WRSS1 shedding^a^** | **53G shedding^a^** | | **Disease Index^b^** | **Bloody stools** | **Loose stools** | **Total stool weight (g)** | | **Clinical symptoms** |
| Vaccinees | | | | | | | | | |
| 1 | Y | | N | **0** | 0 | 1 | 1321 |  | |
| 2 | N | | Y | **0** | 0 | 2 | 1720 |  | |
| 3 | Y | | Y | **2** | 4 | 4 | 276 | Dysentery, nausea, malaise | |
| 4 | Y | | Y | **0** | 0 | 1 | 1335 |  | |
| 5 | N | | Y | **2** | 1 | 9 | 809 | Diarrhea, fever, myalgia, dizziness, anorexia, headache | |
| 6 | N | | N | **0** | 0 | 4 | 894 |  | |
| 7 | Y | | N | **1** | 0 | 0 | 783 | Myalgia, anorexia, malaise, headache | |
| 8 | Y | | N | **2** | 2 | 17 | 2833 | Diarrhea, dysentery, abdominal pain, tenesmus, dizziness, malaise | |
| 9 | N | | Y | **0** | 1 | 0 | 833 |  | |
| 10 | Y | | Y | **0** | 0 | 7 | 1453 |  | |
| Naïve controls | | | | | | | | | |
| 1 |  | | Y | **2** | 1 | 8 | 2304 | Dysentery, fever, tenesmus, anorexia, headache | |
| 2 |  | | N | **2** | 1 | 3 | 426 | Dysentery, abdominal pain, dizziness | |
| 3 |  | | Y | **2** | 0 | 9 | 346 | Diarrhea, nausea, vomit, dizziness | |
| 4 |  | | N | **1** | 0 | 10 | 2569 | Abdominal pain, tenesmus, malaise | |
| 5 |  | | Y | **2** | 0 | 5 | 1983 | Myalgia, malaise | |
| 6 |  | | Y | **0** | 4 | 7 | 1046 |  | |
| 7 |  | | Y | **3** | 6 | 9 | 753 | Diarrhea, dysentery, fever, myalgia, headache | |
| 8 |  | | N | **0** | 0 | 0 | 1566 |  | |
| 9 |  | | Y | **0** | 0 | 0 | 715 |  | |
| 10 |  | | Y | **0** | 0 | 1 | 1510 |  | |
| ^a^Shedding based on culture or PCR of fecal sample  ^b^Disease score was determined as follows: 0 = no clinical symptoms; 1 = no diarrhea, dysentery, and fever, but may have other clinical symptoms; 2 = diarrhea and/or dysentery accompanied by fever, 1 to 2 bloody stools, 1 to 2 loose stools; 3 = diarrhea and/or dysentery accompanied by fever, 3-10 bloody stools, 3-10 loose stools | | | | | | | | | |

**Figure S1. Comparison of US CHIM protection thresholds and antibody levels in Thai adults.** Pre-challenge serum IgG levels in Thai individuals who remained healthy or became sick after *S. sonnei* challenge. Dotted lines indicate optimal protective thresholds identified using ROC analysis of antibody concentrations in volunteers enrolled in a previous US CHIM study (25). Antibody levels were measured with the same method and In-House Standard in both studies.

.
