# Supplementary material for: Protective immunity against Shigella sonnei in vaccinated and challenged adult Thai volunteers
Source: mSphere. 2026 Jul 30;11(8):e00269-26. doi: 10.1128/msphere.00269-26 (PMC13508790; doi:10.1128/msphere.00269-26)
Supplement: Supplementary file 2 [file msphere.00269-26-s0002.docx]

**Supplementary Material Legends**

**Table S1. Geometric Mean Fold Rise (GMFR) of *Shigella* antibodies in serum post-vaccination and challenge.**

**Table S2. GMFR of *Shigella* antibodies in lymphocyte supernatants post-vaccination and challenge.**

**Table S3a. GMFR of *Shigella* antibodies in fecal supernatants post-vaccination.**

**Table S3b. GMFR of *Shigella* antibodies in fecal supernatants post-challenge.**

**Table S4a. GMFR of *Shigella* antibodies in serum post-vaccination and challenge.**

**Table S4b. GMFR of *Shigella* antibodies in lymphocyte supernatants post-vaccination and challenge.**

**Table S4c. GMFR of *Shigella* antibodies post-vaccination in fecal supernatants.**

**Table S4d. GMFR of *Shigella* antibodies post-challenge in fecal supernatants.**

**Table S5. Disease severity scores and clinical symptoms in Thai adults WRSS1 vaccination and subsequent *S. sonnei* 53G challenge.**

**Table S6. Raw antibody concentrations in serum, lymphocyte supernatants, and fecal supernatants.**

**Figure S1. Comparison of US CHIM protection thresholds and antibody levels in Thai adults.** Pre-challenge serum IgG levels in Thai individuals who remained healthy or became sick after *S. sonnei* challenge. Dotted lines indicate optimal protective thresholds identified using ROC analysis of antibody concentrations in volunteers enrolled in a previous US CHIM study (25). Antibody levels were measured with the same method and In-House Standard in both studies.
